# Large language models for whole-learner support: opportunities and challenges

**DOI:** 10.3389/frai.2024.1460364

**Published:** 2024-10-15

**Authors:** Amogh Mannekote, Adam Davies, Juan D. Pinto, Shan Zhang, Daniel Olds, Noah L. Schroeder, Blair Lehman, Diego Zapata-Rivera, ChengXiang Zhai

**Affiliations:** ^1^Department of Computer and Information Science and Engineering, University of Florida, Gainesville, FL, United States; ^2^Department of Computer Science, University of Illinois Urbana-Champaign, Urbana, IL, United States; ^3^Department of Curriculum and Instruction, University of Illinois Urbana-Champaign, Champaign, IL, United States; ^4^School of Teaching and Learning, University of Florida, Gainesville, FL, United States; ^5^Department of Computer Science, University of Oregon, Eugene, OR, United States; ^6^Educational Testing Service, Princeton, NJ, United States

**Keywords:** large language model (LLM), AI and education, non-cognitive aspects of learning, interpretability, pedagogical support of students, educational authoring tool

## Abstract

In recent years, large language models (LLMs) have seen rapid advancement and adoption, and are increasingly being used in educational contexts. In this perspective article, we explore the open challenge of leveraging LLMs to create personalized learning environments that support the “whole learner” by modeling and adapting to both cognitive and non-cognitive characteristics. We identify three key challenges toward this vision: (1) improving the interpretability of LLMs' representations of whole learners, (2) implementing adaptive technologies that can leverage such representations to provide tailored pedagogical support, and (3) authoring and evaluating LLM-based educational agents. For interpretability, we discuss approaches for explaining LLM behaviors in terms of their internal representations of learners; for adaptation, we examine how LLMs can be used to provide context-aware feedback and scaffold non-cognitive skills through natural language interactions; and for authoring, we highlight the opportunities and challenges involved in using natural language instructions to specify behaviors of educational agents. Addressing these challenges will enable personalized AI tutors that can enhance learning by accounting for each student's unique background, abilities, motivations, and socioemotional needs.

## 1 Introduction

In recent years, generative artificial intelligence (GenAI)—and more specifically, LLMs—have exploded into global public awareness (Barreto et al., 2023). ChatGPT, for example, is available in 188 countries with over 180 million users (as of August 2023)^1^. Such rapid adoption and ongoing development continues to disrupt many industries and areas of study, particularly as each new generation of LLMs offers new capabilities (e.g., memory, multimodality, longer input context sizes). LLMs have made their impact in the world of education as well—for instance, one notable example is Khanmigo^2^, an LLM-powered AI tutor that provides personalized support to students and assists teachers with developing instructional materials. This type of personalized support highlights the great potential for LLMs in educational contexts (Pardos and Bhandari, 2023).

We argue that such personalized support systems can and should be further expanded to provide “whole learner” support, moving beyond the paradigm of understanding and supporting only students' *academic proficiency* to also address *social, affective, motivational, cultural*, and *linguistic* characteristics that are known to impact learning (Bernacki et al., 2021; Mercado, 2018; Walkington and Bernacki, 2018; Lehman et al., 2024). This work focuses on “non-cognitive skills,” describing aspects of a learner beyond subject knowledge and proficiency, such as resilience, persistence, empathy, motivation, self-regulation, and a growth mindset (Kautz et al., 2014).

Personalized learning environments (PLEs) leverage learner models, which are structured representations of students, to guide personalized support (Abyaa et al., 2019; Ismail et al., 2023). However, existing learner models cannot support the “whole learner,” as they typically limit themselves to modeling knowledge acquisition (e.g., level of mastery over a concept), and at best, one additional characteristic (e.g., prior knowledge) or behavior (e.g., engagement; Ismail et al., 2023)^3^. The complexity of whole-learner modeling stems from the fact that it is not enough to simply model each characteristic and behavior independently—instead, these factors must be considered holistically to understand and support the whole learner. While the complex interaction of these factors presents a significant challenge for existing PLEs, we know that such holistic support is possible to provide in practice given that human teachers successfully combine these elements to support their students every day.

By pairing whole-learner modeling with GenAI, LLMs present an opportunity to bridge the long-standing gap between the quality and range of learner support offered by present-day computational systems and that offered by expert human tutors (Lepper et al., 1993; Cade et al., 2008; D'Mello et al., 2010). However, rapid innovation in the field of LLMs has raised questions about their appropriate use in PLEs. We explore some of the challenges and opportunities that exist around the vision of using LLMs to build whole-learner models and eventually create adaptive learning systems. We first explore the challenges and potential of LLMs in doing so (Section 2) and then identify several promising research directions to address these challenges (Section 3).

## 2 Challenges and the state of the art

We identify three key areas where the community needs to progress to achieve the larger vision of whole-learner modeling:

- **Interpretable representations of learners:** It is necessary to represent a learner explicitly and faithfully, including both *cognitive* and *non-cognitive* aspects. Although deep learning methods have traditionally been viewed as “black-box” approaches with opaque internal mechanisms, recent advances in interpretability and explainability research are working to address this challenge, and are well-positioned for applications in the context of whole learner modeling and support.
- **Adaptive technologies to support whole learners:** Given an interpretable learner representation, it should be used to tailor the delivery of pedagogical content and support to suit a learner's characteristics, including both cognitive and non-cognitive states. By leveraging data on learner behavior, preferences, and characteristics, and dynamically adjusting instructional strategies to address individual needs, adaptive systems can provide personalized learning pathways that evolve with learners' cognitive and non-cognitive skill development.
- **Authoring and evaluating agents:** In the context of PLEs and pedagogical agents (PAs), the term “authoring” refers to the manual process of specifying behaviors (or “policies") of the agent. For instance, in an intelligent tutoring system (ITS), classroom instructors can *author* common misconceptions based on their teaching experience. Authors can come from varying backgrounds (e.g., researcher, educator, and developer), so a key challenge in designing authoring tools is balancing accessibility and minimizing cognitive load. Finally, authoring is tightly-coupled with the issue of evaluation, a critical step in smoothly deploying these systems to real learners.

### 2.1 Interpretable representations of learners

Two key concerns in deploying LLMs to potentially sensitive application contexts such as education are *interpretability* (what a “black box” model is doing and representing internally) and *explainability* (why the model outputs A instead of B, given input C). Without reliable *interpretation*, we do not know what information the models use to make decisions or generate responses. Unlike trained educational professionals, automated models cannot be trusted to reliably take students' prior knowledge or emotional state into account to provide relevant and compassionate guidance, nor can we be certain that they will not use sensitive demographic information inappropriately. On the other hand, when we cannot reliably *explain* LLM behaviors, we cannot ensure that desired behaviors in one context will generalize to others (e.g., whether attentiveness to the emotional needs of students with high socioeconomic status will translate to less advantaged students).

Integrating interpretable learner models with LLMs is a promising approach to develop PLEs, providing the benefits of GenAI while maintaining a high level of interpretability. Such a hybrid approach need not be overly complex; for instance, one may begin by training a traditional learner model and passing its inferences to the LLM as an additional component of input prompts. However, it is crucial to ensure that (1) LLMs actually consider learner model's output, and that (2) they use this information in a way that is faithful to the learner model and consistent with educational best practices – otherwise, the approach will not benefit educational stakeholders like teachers and students (Pinto et al., 2023).

LLMs are also well-suited for advancing open learner models (OLMs) due to their natural language dialogue capabilities. OLMs enable learners to view and interact with the model's representation of their knowledge, promoting reflection and self-regulated learning. This transparency and interactivity can enhance traditional OLMs, allowing learners to modify their learning paths more freely. However, the use of LLMs in OLMs also raises concerns about how LLMs use learner information and relate their actions to educational best practices. Indeed, the integration of LLMs with OLMs has the potential to revolutionize educational technology by making learning processes more adaptive and personalized (Conati et al., 2018; Kay et al., 2020; Zapata-Rivera and Arslan, 2021; Bull, 2020), but implementation must be guided by strong ethical and pedagogical standards.

Despite important recent advances in understanding the inner workings of LLMs (e.g., Elhage et al., 2021; Olsson et al., 2022; Conmy et al., 2023; Templeton et al., 2024), reliably explaining model behavior to relevant stakeholders remains a significant challenge. This inability to interpret LLM representations and explain model behaviors leads to a lack of trust (Shin, 2021; Liao and Vaughan, 2023), which can inhibit these models' deployment to educational contexts where they have potential for transformational impact. By contrast, many traditional learner models are designed with interpretability as an inherent feature, such as Bayesian Knowledge Tracing (Baker et al., 2008) and Item Response Theory (Yen and Fitzpatrick, 2006). There are even efforts underway to develop intrinsically interpretable neural-network-based learner models (Pinto et al., 2023; Lu et al., 2020; Swamy et al., 2024). We discuss how such approaches can address the challenges of interpretable LLMs for education in 3.1.

### 2.2 Whole learner support through adaptive technologies

The next challenge in deploying LLMs for education is *adaptivity*, which involves assessing various learner characteristics and tailoring the learning experience to individual needs to improve learning outcomes (Plass and Pawar, 2020). Through the natural language capabilities of LLMs, adaptive technologies for whole learner support can offer nuanced support for developing both cognitive and non-cognitive skills in diverse learners. For instance, Arroyo et al. (2014) demonstrated that intelligent adaptive tutors effectively address students' unique needs and emotions, enhancing engagement and affect, while Liu et al. (2024) found that conversational agents offering emotional scaffolding improved students' emotional experiences.

Such findings highlight the importance of design principles focused on non-cognitive learner characteristics, such as fostering a growth mindset through praising learners' efforts (Liu et al., 2024), attributing struggles to external factors (Calvo and D'Mello, 2011), utilizing an anthropomorphic language style, and employing proactive inquiry (McQuiggan et al., 2008; Sabourin et al., 2011) to guide learners to self-report their emotional states. For instance, a review found that empathetic agent feedback, including affective feedback and confidence- and motivation-enhancing dialogue, positively influences students' attitudes (Liu et al., 2024; Ortega-Ochoa et al., 2024). Similarly, another study demonstrated how conversational agents can support children's social-emotional learning by teaching self-talk. These lines of research also emphasize the importance of designing conversational dialogue based on an evidence-based framework (Fu et al., 2023). Building on this foundation, recent AI advancements have facilitated the development of natural language dialogue systems to scaffold non-cognitive skills (Acosta et al., 2015; Anghel and Balart, 2017; Cinque et al., 2021).

### 2.3 Authoring and evaluating agents

LLMs are also transforming the landscape for authoring educational agents such as PAs, intelligent tutors (Sottilare et al., 2015), and even simulated learners (Käser and Alexandron, 2023). Before the widespread adoption of modern LLMs, agent authoring was bottlenecked by supervised and reinforcement learning methods that required machine learning expertise (Mannekote et al., 2023; Liu and Chilton, 2022), lots of data, labor-intensive manual annotation, or some combination of these factors. In contrast, the recent development of instruction-tuned LLMs (Wang et al., 2023) enables educational experts to define agent behaviors using natural language instructions in “zero-shot” or “few-shot” setups (i.e., using no annotated examples or only a few, respectively). In addition to reducing the training and expertise needed for authoring the dialogue system, LLMs also open up new avenues of agent behavior—for instance, where classical ITSs predominantly focused on supporting the cognitive aspects of learning (e.g., subject proficiency) (Sottilare et al., 2015), authors can now leverage LLMs capabilities such as their abilities to emulate human-like decision-making (Miliv cka et al., 2024) and perform high-level planning (Kambhampati et al., 2024) to equip them with the ability to support non-cognitive aspects of learning as well.

However, LLMs are not (yet) a “turn-key” solution to agent authoring, as several key challenges remain. Authoring LLM-based agents requires effectively navigating an unbounded space of possible prompts, which may be difficult to do without prompt engineering expertise (Oppenlaender et al., 2023; Zamfirescu-Pereira et al., 2023; Mannekote et al., 2023). Moreover, it has been shown that LLM outputs are highly sensitive to minor prompt variations, often leading to inconsistent (Lu et al., 2022; Liu and Chilton, 2022; Loya et al., 2023; Mohammadi, 2024) and confounding (Gui and Toubia, 2023) results. Finally, when authoring complex agent behaviors, the issue of evaluating the *faithfulness* of an agent's behavior to the authors' intended expectations becomes pertinent (Koedinger et al., 2015; Weitekamp et al., 2023). In fact, within the context of AI models like LLMs, this issue can be considered to be a specific instance of the *alignment problem* (Yudkowsky, 2016).

## 3 Ways forward

For each of the three challenge areas delineated in Section 2, we outline a broad roadmap for future advancements. Specifically, we identify promising directions that the field is likely to pursue in the medium to long term.

### 3.1 Interpretable representations of learners

We face two primary challenges in enhancing the interpretability of LLMs. First, rather than merely adding more information to the prompt and hoping that the model will use it appropriately, we need a direct method to explain why a model generated a particular output. This involves determining whether the output was produced due to explicit learner information that has been added to prompts or implicit learner information that LLMs have inferred from learner behavior. Second, we must predict whether its behavior will remain consistent when applied in different contexts, such as in learning environments for which they have not already been tested. Although neural networks like LLMs are usually seen as “black boxes” whose internal representations and mechanisms are treated as unknowable beyond the outputs they produce, recent work in deep learning interpretability has made substantial strides in addressing this challenge. For instance, current interpretability methods can detect what latent representations are used by models in producing a particular output (Elazar et al., 2021; Belinkov, 2022; Davies et al., 2023) and characterize how these representations are leveraged in producing particular behaviors (Elhage et al., 2021; Olsson et al., 2022; Conmy et al., 2023).

Beyond simply interpreting LLMs' representation and use of information about learners, it is also important to utilize counterfactual explanation techniques to predict how their behaviors will change in response to different input prompts (Wachter et al., 2017; Ribeiro et al., 2020)—for instance, if we add a minor typo to a student's essay that is otherwise exemplary, will the model provide a substantially lower assessment of the student's knowledge in response? Conversely, it is equally important to characterize when and how models will remain invariant with respect to a given input property (Schwab and Karlen, 2019)—for example, it may be important to understand whether models always provide the same answer to different ways of phrasing the same question, meaning that they are *invariant* with respect to question semantics. Knowing the set of properties to which a given model is invariant allows us to predict whether its behavior will remain consistent if those same properties are held constant, even as other input properties may vary (Peters et al., 2016; Arjovsky et al., 2019). Between these two lines of research, we can build a systematic picture of when, how, and why model behaviors are expected to change (under counterfactuals) or remain the same (given invariances).

### 3.2 Whole learner support through adaptive technologies

Our vision for personalized learning that supports whole-learner adaptation necessitates a dynamic approach to learner modeling, capable of capturing and integrating the learner's complex states and needs. High-quality adaptive feedback is contingent on an accurate representation of the learner. Crafting and updating this representation is the job of the learner model, which is typically considered a separate component from the adaptation module that produces feedback in ITSs and PLEs (Shute and Zapata-Rivera, 2008). While learner models can come in many forms, such as cognitive models, machine learning models, or Bayesian networks, GenAI models like LLMs are beginning to be tested for this task (Zhang et al., 2024).

Integrating whole learner models with LLM-based support involves using cognitive, affective, or behavioral states from learner models as inputs to the adaptation module or dialogue engine (Zapata-Rivera and Forsyth, 2022). To capture the whole learner, multiple traditional models representing distinct aspects of the learner can either form a larger learner *module* or be integrated into a holistic model. Alternatively, a single LLM might serve as both the learner model and the adaptation module, though the current lack of LLM interpretability challenges trustworthiness and validation. Another viable option is leveraging a LLM to integrate outputs from various traditional learner models, providing a comprehensive inference to the adaptation module. This approach could be very useful, despite the limited research on integrating diverse types of learner information, as it offers a more nuanced understanding of the student.

Regardless of the specific system architecture used, LLMs enable just-in-time adaptive conversational feedback. This allows conversational complexity to adjust dynamically based on the learner's real-time progress, maintaining an appropriate level of challenge and promoting engagement (Zapata-Rivera and Forsyth, 2022). By basing this feedback on a rich understanding of the learner from the learner model, it offers whole-learner adaptation, potentially providing more nuanced, personalized support than existing PLEs.

### 3.3 Authoring and evaluating agents

When building agents to support the whole learner, the ability to operationalize a given theoretical model or dynamically incorporate new developments from learning sciences into agentic behavior “on the fly” is a desirable trait, helping to avoid the tedious process of manually re-authoring the agents. Efficient attention mechanisms (Shen et al., 2021), attention-alternatives (Gu and Dao, 2023), techniques such as retrieval-augmented generation (RAG) (Lewis et al., 2020), and needle-in-the-haystack capabilities (Kuratov et al., 2024) will enable authors to quickly reshape agent behavior, potentially even allowing them to directly operationalize longer documents such as scientific reviews or books describing evidence-based practices.

Equally important to authoring is evaluating the model outputs for faithfulness and robustness. Although preliminary experimental results with using LLMs in economics and psychology suggest that LLMs are capable of accurately mimicking aspects of human behavior like decision-making (Jia et al., 2024) and personality traits (Frisch and Giulianelli, 2024), further research is needed to generalize these findings to educational settings.

Finally, authoring is not just about *designing* agents for pedagogical support, but also developing realistic testbeds to *evaluate* them. For this line of work, authoring multi-agent social simulations (see, e.g., Park et al., 2022, 2023) will be an integral component of the end-to-end development process of ITSs and PAs. Such evaluations can ensure that the agents perform well across a wide range of scenarios, increasing educator confidence. For instance, instead of testing a PA against a single learner simulation, authoring an entire classroom of LLMs comprising multiple learner agents allows for more holistic and rigorous testing, ensuring the PA is pedagogically effective, equitable, safe, and robust before being deployed to real learners.

## 4 Ethical considerations

Several important ethical considerations must be addressed before deploying GenAI for educational applications. First, interpretability is crucial for trustworthiness: one cannot fully trust a model in sensitive applications like education without understanding how it represents and interacts with users (Huang et al., 2020). Second, it is important to ensure that LLMs do not exacerbate the *digital divide* in education (i.e., inequitable access to educational technologies and associated benefits), as anticipated by Capraro et al. (2024). For instance, given the substantial compute required to deploy the largest and most capable LLMs, it may be helpful to develop more compute-efficient language models for use in educational settings with limited resources (Hoffmann et al., 2022); and interdisciplinary collaborations between AI research and learning sciences will be essential in ensuring that new technologies are actually improving learning outcomes and student welfare (cf. Dahlin, 2021).

Finally, perhaps most important are concerns regarding student privacy—for instance, in the adaptive support modules envisioned above, LLMs might be provided with information about learners' emotional states to provide more holistic, empathetic feedback; but in order to protect students' privacy and ensure that sensitive information about them cannot be used for non-educational purposes such as advertising, student data should only be visible to systems with robust security and data privacy guarantees (and not, e.g., included in prompts used as input to third-party AI systems, which may use such information to train future public-facing models). These concerns are particularly significant for minors, who have special legal privacy protections and may be more vulnerable to unintended GenAI behaviors.

## 5 Conclusion

In this paper, we explored the potential integration of LLMs into PLEs to support the whole learner addressing both cognitive and non-cognitive characteristics. Our discussion has highlighted significant opportunities as well as challenges in integrating LLMs into PLEs, focusing on developing interpretable learner representations, adaptive technologies for personalized support, and authoring and evaluating PAs. For future research, it will be important to develop methods to enhance LLM interpretability and explainability within educational settings, facilitating trustworthiness and appropriate use of student information. Additionally, LLMs' adaptability must also be refined to ensure that models can offer individualized support that accounts for diverse learner needs and backgrounds. Finally, authoring PAs will require more principled prompting protocols, including an understanding of both relevant subject matter and pedagogical best practices, in order to engender more faithful and robust agents. By advancing each of these areas, LLMs can be better positioned to fulfill their potential as transformative tools in education, making widely-accessible personalized learning a practical reality. Through all these advancements, it is essential to be mindful of the security, privacy, and ethical concerns surrounding the handling of learner data.

## Data Availability

The original contributions presented in the study are included in the article/supplementary material, further inquiries can be directed to the corresponding author.

## Footnotes

^1^ https://clickup.com/blog/chatgpt-statistics/#6-chatgpt-users-and-usage-

^2^ https://blog.khanacademy.org/harnessing-ai-so-that-all-students-benefit-a-nonprofit-approach-for-equal-access/

^3^However, models considering more learner characteristics have been proposed (Shute and Zapata-Rivera, 2008; Zapata-Rivera and Greer, 2004).
